# Supplementary material for: Erdafitinib Inhibits Tumorigenesis of Human Lung Adenocarcinoma A549 by Inducing S-Phase Cell-Cycle Arrest as a CDK2 Inhibitor
Source: Molecules. 2022 Oct 9;27(19):6733. doi: 10.3390/molecules27196733 (PMC9573074; doi:10.3390/molecules27196733)
Supplement: Supplementary file 1 [file molecules-27-06733-s001.zip › molecules-1870292-supplementary.pdf]

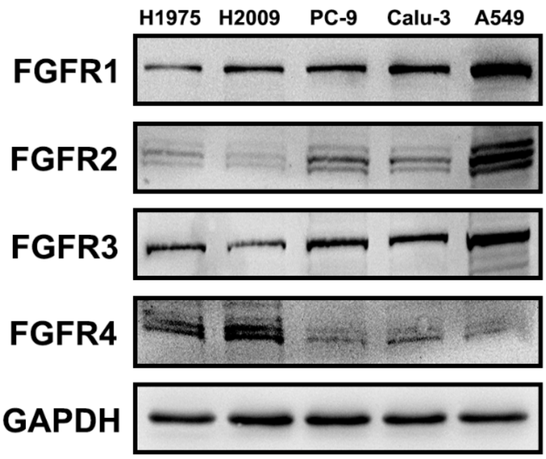

Figure S1. The expressions of FGFR 1-4 in human lung adenocarcinoma cell lines including A549, H1975, H2009, Calu-3 and PC-9 were assessed by Western blot analysis.

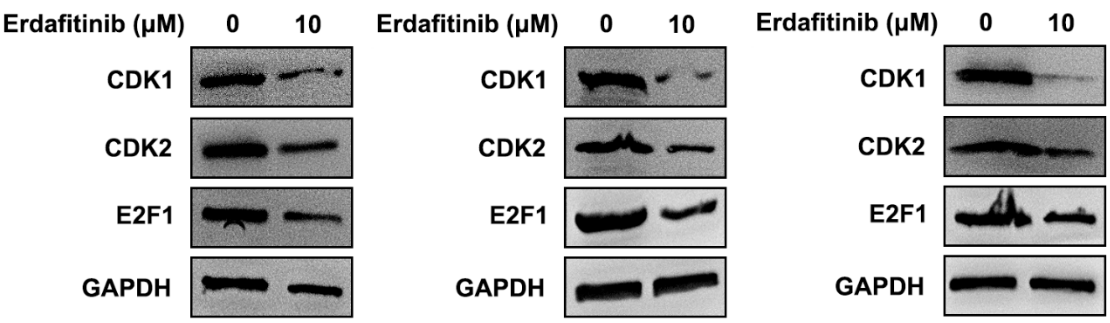

Figure S2. The protein expressions of CDK1, CDK2 and E2F1 were assessed by Western blot analysis in cells with or without erdafitinib treatment (10 μM, 24 h).

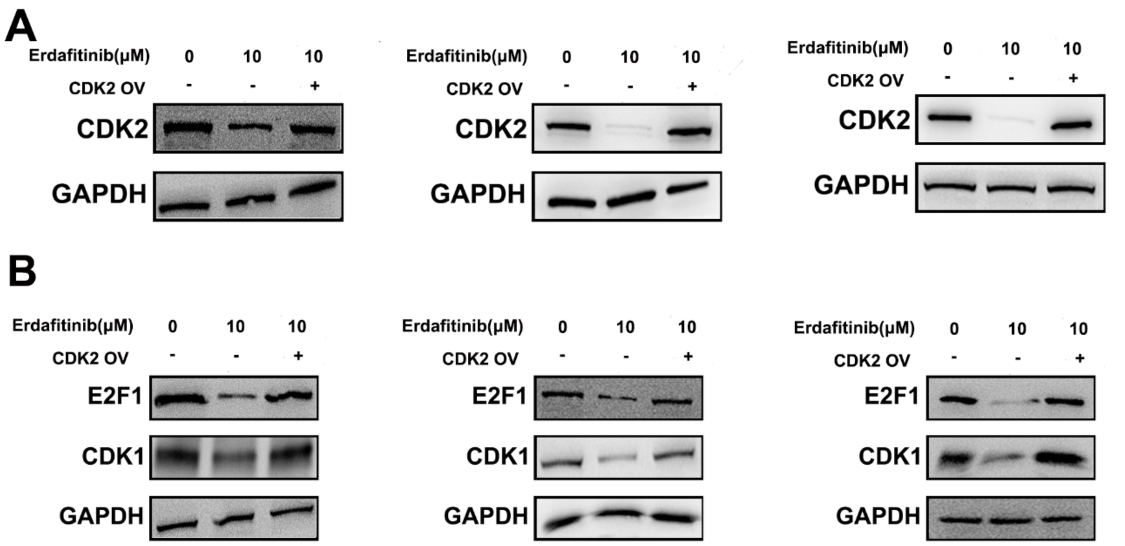

Figure S3. A549 cells were transfected with pcDNA3.1-CDK2 for 48 h and then treated with erdafitinib (10 μM) for 24 h. (A) The expression of CDK2 was assessed by Western

blot analysis. (B) The expressions of E2F1 and CDK1 were assessed by Western blot analysis.

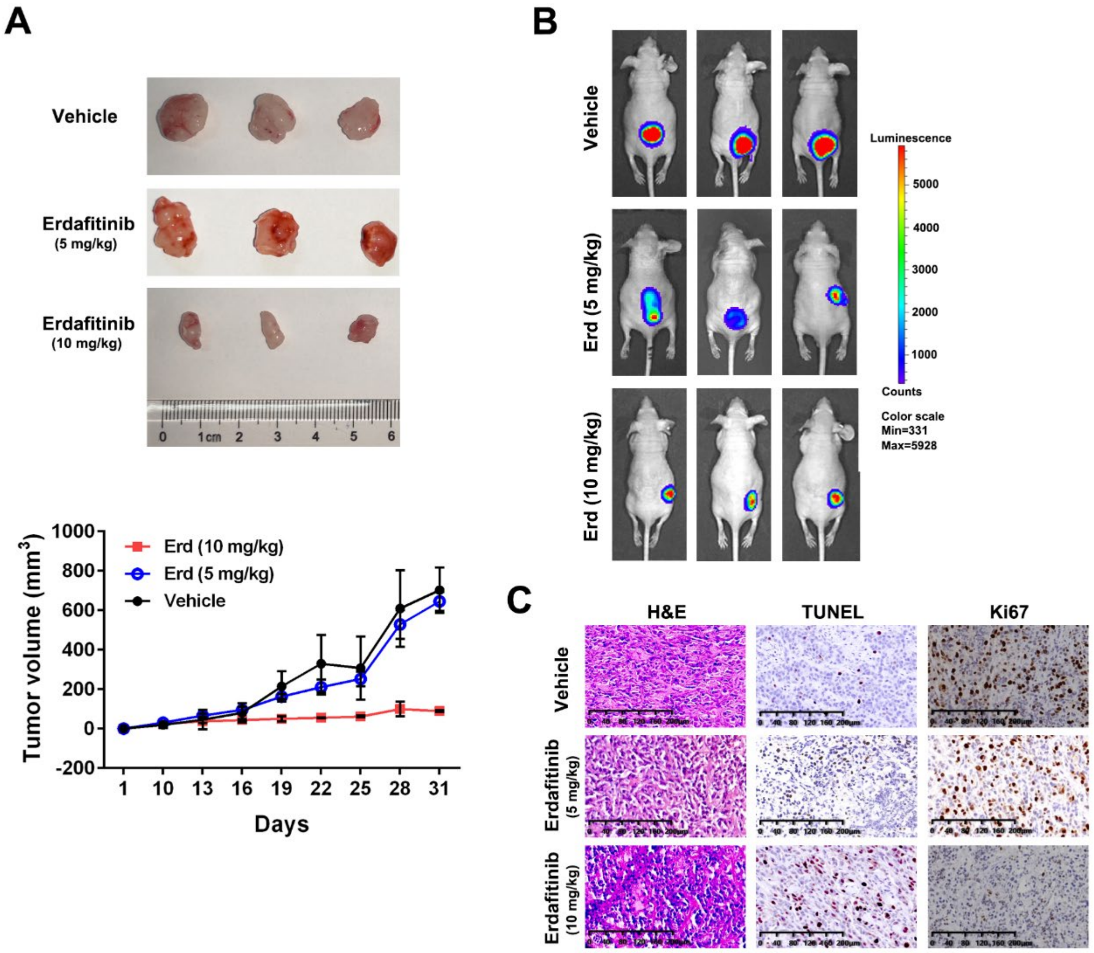

Figure S4. Erdafitinib suppresses tumor growth in a A549 xenograft mice model. Nude mice were intraperitoneally injected with vehicle or erdafitinib (5 or 10 mg/kg/day) for 21 days. **(A)** Tumor volumes were measured every other day. At the end of the treatment, the tumors were removed and photographed (n=3). The images of tumors were shown on the up panel and the tumor growth curve was displayed on the down panel. **(B)** Live imaging of the animals prior to euthanasia. **(C)** Representative images of hematoxylin-eosin (H&E), TUNEL and Ki67 staining in A549 xenografts. Data was expressed as mean ± SD of three experiments.
